# Influence of *UGT1A1* and *SLC22A6* polymorphisms on the population pharmacokinetics and pharmacodynamics of raltegravir in HIV-infected adults: a NEAT001/ANRS143 sub-study

**DOI:** 10.1038/s41397-022-00293-5

**Published:** 2022-10-20

**Authors:** Rohan Gurjar, Laura Dickinson, Daniel Carr, Wolfgang Stöhr, Stefano Bonora, Andrew Owen, Antonio D’Avolio, Adam Cursley, Nathalie De Castro, Gerd Fätkenheuer, Linos Vandekerckhove, Giovanni Di Perri, Anton Pozniak, Christine Schwimmer, François Raffi, Marta Boffito, the NEAT001/ANRS143 Study Group

**Affiliations:** 1 Department of Pharmacology & Therapeutics, University of Liverpool, Liverpool, UK; 2 MRC Clinical Trials Unit at UCL, London, UK; 3 Unit of Infectious Diseases, University of Turin, Turin, Italy; 4 Infectious Diseases Department, AP-HP Hôpital Saint-Louis, Paris, France; 5 Unit of Internal Medicine, University Köln, Köln, Germany; 6 HIV Translational Research Unit, Ghent University and Ghent University Hospital, Ghent, Belgium; 7 Chelsea and Westminster NHS Trust, London, UK; 8 University of Bordeaux, INSERM, Bordeaux Population Health Research Center, UMR 1219, Bordeaux, France; 9 Department of Infectious Diseases, Centre Hospitalier Universitaire de Nantes, and CIC 1413, INSERM, Nantes, France; 10 Imperial College, London, UK

**Keywords:** Pharmacogenetics, Pharmacokinetics, Clinical pharmacology

## Abstract

Using concentration-time data from the NEAT001/ARNS143 study (single sample at week 4 and 24), we determined raltegravir pharmacokinetic parameters using nonlinear mixed effects modelling (NONMEM v.7.3; 602 samples from 349 patients) and investigated the influence of demographics and SNPs (*SLC22A6* and *UGT1A1*) on raltegravir pharmacokinetics and pharmacodynamics. Demographics and SNPs did not influence raltegravir pharmacokinetics and no significant pharmacokinetic/pharmacodynamic relationships were observed. At week 96, UGT1A1*28/*28 was associated with lower virological failure (*p* = 0.012), even after adjusting for baseline CD4 count (*p* = 0.048), but not when adjusted for baseline HIV-1 viral load (*p* = 0.082) or both (*p* = 0.089). This is the first study to our knowledge to assess the influence of SNPs on raltegravir pharmacodynamics. The lack of a pharmacokinetic/pharmacodynamic relationship is potentially an artefact of raltegravir’s characteristic high inter and intra-patient variability and also suggesting single time point sampling schedules are inadequate to thoroughly assess the influence of SNPs on raltegravir pharmacokinetics.

## Introduction

Raltegravir was the first integrase inhibitor approved for the treatment of HIV-1. Safety and efficacy of raltegravir have been demonstrated in treatment-experienced (BENCHMARK 1 and 2) [1] and treatment-naïve (STARTMRK) [2] patients and is recommended for initial therapy in numerous guidelines [3, 4]. Initially dosed at 400 mg twice daily, it is also available as a new 600 mg formulation; 2 pills once daily for first-line therapy [5].

Raltegravir is unique since it is metabolised through glucuronidation by uridine diphosphate glucuronosyltransferase 1A1 (UGT1A1) with no involvement of cytochrome P450 (CYP450) enzymes [6]. Additionally, raltegravir does not alter CYP450 activity [7] making it less prone to drug-drug interactions and safe to co-administer with CYP450 substrates [8]. Furthermore, raltegravir is generally well tolerated and has a low incidence of adverse events causing treatment discontinuation [9]. Notwithstanding its advantages, raltegravir displays a broad inter-subject and intra-subject variability [10] and has a low genetic barrier to drug resistance [11]. This has complicated pharmacokinetic/pharmacodynamics (PK/PD) analysis and made it difficult to estimate PK thresholds for efficacy and toxicity [12].

The influence of *UGT1A1* polymorphisms on the PK and efficacy of raltegravir has been a matter of dispute, owing to numerous studies producing variable results [10, 13–17]. Additionally, raltegravir is a substrate of SLC22A6 (OAT1) [18] and polymorphisms in the *SLC22A6* gene [19] could influence raltegravir disposition. We investigated the population pharmacokinetics (popPK) of raltegravir 400 mg twice daily and the influence of demographic covariates and polymorphisms in the *UGT1A1* and *SLC22A6* genes on raltegravir treatment response in patients randomised to the ritonavir-boosted darunavir (800/100 mg once daily) plus raltegravir arm in the Phase III NEAT 001/ANRS 143 study [20].

## Results

### Patients and pharmacokinetic sampling

**Fig. 1:**
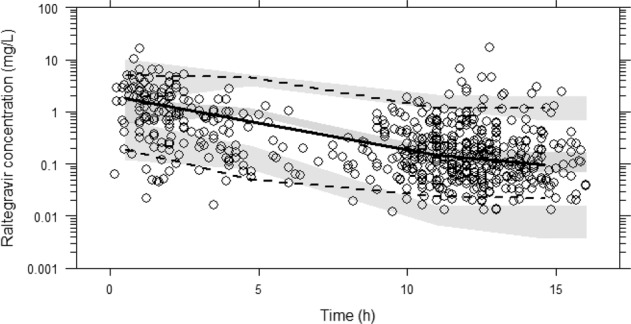
Raltegravir visual predictive check (VPC). The lines represent the percentiles of the observed data (P5, P50, P95) and the shaded areas the 95% CI of the simulated data. Observed raltegravir concentration-time data (*n* = 349 patients, 602 concentrations) are superimposed (open circles).

Of 401 patients randomised to the raltegravir arm, 386 (*n* = 726 samples) provided data for this analysis. In total 602 samples (*n* = 313 week 4, *n* = 289 week 24) from 349 patients receiving raltegravir 400 mg twice daily were used for model development. A total of 124 samples (17.1%) were excluded due to missing time post-dose, missing concentration, time post-dose greater than 16 h, plasma raltegravir below the bioanalytical assay lower limit of quantification (LLQ) or a mixture of the above. Raltegravir concentrations ranged between 0.012 and 17.3 mg/L sampled 0.17–16.0 h post-dose (Fig. 1). Patient characteristics are described (Table 1). Patients excluded from the PK modelling (*n* = 52) had similar characteristics apart from country and HIV-RNA (4.55 log_10_ copies/mL in those excluded).

**Table 1:** Clinical characteristics, demographics and genetic polymorphisms of patients included in the population pharmacokinetic model of raltegravir for the NEAT001/ANRS143 pharmacokinetic sub-study [data expressed as median (range) unless stated otherwise].

Parameter	
Included for modelling (*n*)	349
Sex [*n* (%)]	
Male	306 (87.7)
Female	43 (12.3)
Age (years)	37 (20–71)
Weight (kg)	72 (41–135)
CD4 + T cell count (cells/ mm^3^)	340 (5–780)
HIV-RNA (log_10_ copies/mL)	4.82 (3.11–6.31)
Ethnicity [*n* (%)]	
Caucasian	288 (82.5)
Black	44 (12.6)
Asian	8 (2.3)
Other	9 (2.6)
*SLC22A6* 453G>A (rs4149170) [*n* (%)]	
GG	216 (61.9)
GA	68 (19.5)
AA	9 (2.6)
Missing	56 (16.0)
*SLC22A6* 728C>T (rs11568626) [*n* (%)]	
CC	285 (81.7)
CT	6 (1.7)
TT	2 (0.6)
Missing	56 (16.0)
UGT1A1*28 (rs8175347) [*n* (%)]	
*1/*1, *1/*36 (normal enzyme activity)	109 (31.2)
*1/*28, *28/*36, *36/*37^a^ (reduced enzyme activity)	115 (33.0)
*28/*28^a^ (low enzyme activity)	40 (11.5)
*36/*36^b^ (unknown enzyme activity)	1 (0.3)
Missing	84 (24.1)

^a^Reduced enzyme activity consists of genotype *1/*6, *1/*28, *1/*37, *28/*36, *36/*37 and low enzyme activity consists of *28/*28, *28/*37, *37/*37. Note that no patients in this cohort had the *1/*6 or *1/*37 genotypes (reduced) or *28/*37 or *37/*37 genotypes (low).

^b^The impact of *36/*36 on UGT1A1 enzyme activity is unknown and therefore was excluded from the population pharmacokinetic covariate analysis.

### Genotyping

Fifty-six patients did not have a blood sample drawn for genotyping; 84.0% (293/349) had both PK and genetic data for *SLC22A6* 453G>A and *SLC22A6* 728C>T whereas genotype was not available in an additional 28 patients for UGT1A1*28 (265/349, 75.9% with data). One patient possessing UGT1A1*36/*36 was excluded from the pharmacogenetic analysis due to the unknown impact of this allele [21]. Genotypes did not deviate from Hardy–Weinberg equilibrium and allele frequencies are summarised in Table 1.

### Population pharmacokinetic modelling

Raltegravir was described by a two-compartment model with first-order absorption parameterised by apparent oral clearance (CL/F), apparent volume of distribution of the central and peripheral compartments (*V*_c_/*F* and *V*_p_/*F*, respectively), intercompartmental clearance (Q/F) and absorption rate constant (*k*_a_); priors from the literature were used for all fixed effects with the exception of raltegravir CL/F [22]. Interindividual variability was included on CL/F and a proportional error model described residual variability.

**Fig. 2:**
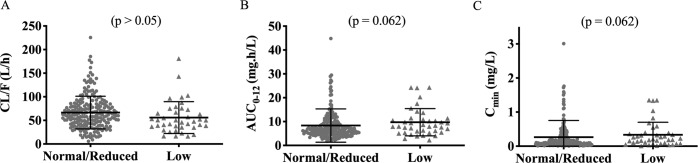
Scatter plots of raltegravir pharmacokinetic parameters stratified by UGT1A1 activity. Mean and SD of raltegravir **A** apparent oral clearance (CL/F), **B** exposure (AUC_0–12_) and **C** minimum concentration (*C*_min_; concentration 12 hours post-dose) in patients with low UGT1A1 activity compared to patients with normal and reduced UGT1A1 activity combined (*p* > 0.05).

None of the covariates evaluated (weight, age, sex, ethnicity, and polymorphisms in *UGT1A1* and *SLC22A6)* produced statistically significant decreases in objective function value (OFV) and therefore a multivariable analysis was not possible. Changes in OFV resulting from the univariable addition of covariates into the model along with corresponding mathematical descriptions are summarised as Supplementary material (Supplementary Table S1). Of note, despite the lack of statistical significance, raltegravir CL/F was reduced by 21% (Fig. 2A) in patients with low UGT1A1 activity compared to those with normal/reduced activity (reference population; normal and reduced combined due to ≤10% difference in population CL/F values between the two groups). Fixed and random effects obtained for the final raltegravir model and visual predictive check (VPC) are presented (Table 2 and Fig. 1). Goodness-of-fit plots are also shown (Supplementary Fig. S1).

**Table 2:** Population pharmacokinetic parameter estimates and relative standard errors (RSE) for the final raltegravir model (*n* = 349 individuals). Of the covariates tested none fulfilled the statistical criteria to remain in the model.

Parameter	Estimate (RSE%)
Fixed effects	
CL/F (L/h)	55.8 (4.1)
*V*_c_/*F* (L)	194 (6.5)
*Q*/*F* (L/h)	13.0 (4.0)
*V*_p_/*F* (L)	117 (0.6)
*k*_a_ (h^−1^)	1.12 (13.0)
Random effects	
IIV CL/F (%)	62.7 (12.1)
Residual error	
Proportional (%)	69.9 (7.0)

RSE = (SE_ESTIMATE_/ESTIMATE) × 100.

*CL/F* apparent oral clearance, *V*_*c*_*/F* apparent volume of distribution of the central compartment, *Q/F*  intercompartmental clearance, *V*_*p*_*/F* volume of the peripheral compartment, *k*_a_ absorption rate constant *IIV*, interindividual variability.

Predicted mean (±s.d.; CV%) raltegravir AUC_0–12_, *C*_max_, C_12_ and half-life were 8.70 mg.h/L (8.20; 94%), 1.44 mg/L (0.68; 47%), 0.29 mg/L (0.60; 205%) and 9.13 h (3.94; 43%), respectively; median (range) *T*_max_ was 1.50 h (1.00–2.00). Substantial interindividual variability was observed in the C_12_ estimates. A post-hoc analysis was performed to determine the impact of UGT1A1*28 on predicted raltegravir AUC_0–12_ and C_12_ [low activity (*n* = 40) vs. normal/reduced activity as reference (*n* = 224)]. Geometric mean ratio (90% CI), back-transformed from log values were 1.34 (0.99–1.84; *p* = 0.062) and 1.32 (0.99–1.77; *p* = 0.062) respectively, suggesting a modest increase, although not statistically significant, in AUC_0–12_ and C_12_ of patients with low activity *UGT1A1* genotype compared to the reference genotype (Fig. 2B, C).

### Pharmacokinetic–pharmacodynamic analysis

The analysis of raltegravir PK parameters included 349 patients of which 58 had virological failure (VF; 16.6%). We found no significant association of raltegravir C_12_ or AUC_0–12_ with time to VF overall (multivariable HR: 0.72 per log_10_ mg/L increase (95% CI 0.44–1.17), *p* = 0.181; and 0.48 per log_10_ mg.h/L increase (95% CI 0.17–1.38), *p* = 0.173, respectively). Results were similar when censoring after switch from randomised regimen, after multiple imputation of missing PK parameters or when analysing time to the trial primary endpoint (results not shown). Similarly, we did not see an association between raltegravir PK parameters and change in CD4 cell count from baseline (C_12_: −1.3 (95% CI −41.0 to 38.4) cells/mm^3^ per log_10_ mg/L increase, *p* = 0.940; AUC_0–12_: −0.6 (95% CI −77.9 to 76.7) cells/mm^3^ per log_10_ mg.h/L increase, *p* = 0.99).

### Adverse events

Thirty-two of 349 participants (9.2%) experienced grade 2 or higher triglycerides by week 96, and we found a higher risk with higher raltegravir AUC_0–12_ (HR 6.24 per log_10_ mg/L increase; 95% CI 1.88 to 20.72; *p* = 0.003). Fifty participants had creatine kinase grade 2 or higher, however, there was no association with raltegravir AUC_0–12_ (HR 1.11 per log_10_ mg/L increase; 95% CI 0.42 to 2.98; *p* = 0.83). There also was no association between raltegravir AUC_0–12_ and LDL levels post-randomisation (−0.10 mmol/L (95% CI −0.33 to 0.13) per log_10_ mg/L increase; *p* = 0.39). Similarly, no significant associations were seen with raltegravir *C*_max_ (results not shown).

### Integrase resistance

Fifteen of 349 participants experienced VF with integrase resistance mutations by week 96. We found no significant association of raltegravir C_12_ or AUC_0–12_ with time to detection of integrase resistance mutations (HR, adjusted for baseline CD4 and HIV-1 viral load: 0.70 per log_10_ mg/L increase (95% CI 0.42–1.16), *p* = 0.163; and 0.17 per log_10_ mg.h/L increase (95% CI 0.01–2.07), *p* = 0.164, respectively).

### Genetic association analysis

**Fig. 3:**
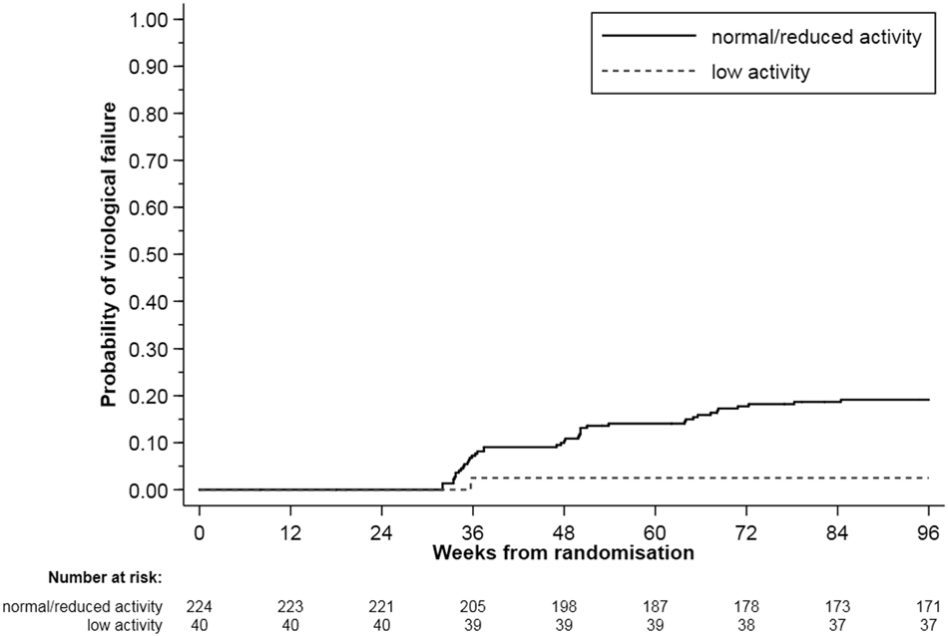
Kaplan-Meier plot of multivariable Cox regression analysis. Probability of virological failure in patients with low UGT1A1 activity compared to normal and reduced UGT1A1 activity combined at week 96. (*p* = 0.012).

In the 264 participants assessed, a significantly lower incidence of VF by week 96 was seen in patients with low UGT1A1 activity (1 failure; cumulative risk 2.5%) compared to those with normal/reduced activity (42 failures; 19.2%), *p* = 0.012 (Fig. 3). This association remained significant after adjusting for baseline CD4 count (HR = 0.14 [95%CI 0.02–0.99] *p* = 0.048), but not when adjusted for baseline HIV-1 viral load (HR = 0.17; *p* = 0.082) or both baseline CD4 and HIV-1 viral load (HR = 0.18; *p* = 0.089).

Integrase resistance mutations were detected in 1/40 (cumulative risk 2.6%) participants with low UGT1A1 activity versus 13/224 (6.3%) in patients with normal/reduced activity (unadjusted HR 0.39; *p* = 0.363; adjusted for baseline CD4 and HIV-1 viral load: HR 0.83; *p* = 0.856). We did not find any association between UGT1A1*28 genotype and any of the adverse events (results not shown).

## Discussion

We developed a popPK model of raltegravir, administered as 400 mg twice daily, using data from the NEAT001/ANRS143 study [20]. Raltegravir was best described by a 2-compartment model with first-order absorption but both 1- and 2-compartment models have been reported [22, 23]. The estimated mean AUC_0–12_, *C*_max_ and C_12_ were comparable to those achieved in the phase III QDMRK study, with raltegravir 400 mg twice daily [24]. Similarly, raltegravir *T*_max_ and half-life were in line with literature values [25] and estimated CL/F was within the range of previous popPK studies, although reported estimates are highly variable (e.g. 39.1, 60.2, 80.6 L/h) [22, 23, 26]. Considerable interindividual variability was observed (CL/F:62.7%, C_12_:205%), which is expected with raltegravir.

Sex-related differences such as higher gastric pH and lower P-glycoprotein (P-gp) expression in females [27], ethnicity-related differences due to variable plasma protein binding and P-gp expression [28], and age-related changes such as reduced renal and hepatic clearance [29] can potentially influence raltegravir PK. However, the clinical effects of such differences have not been observed in most studies [26, 30]. Similarly, in our study, sex, weight and ethnicity did not influence raltegravir CL/F. In contrast, a popPK analysis showed a 55% higher raltegravir relative bioavailability in females and a 65% lower V/F in Caucasians, however this contributed little to the reduction in parameter variability [22].

Raltegravir has a marked inter and intraindividual variability, especially with *C*_min_ concentrations [10], complicating PK/PD analyses_._ In the QDMRK study, a correlation between *C*_min_ and viral suppression was observed in patients receiving raltegravir 800 mg once daily, but not in patients receiving 400 mg twice daily [24]. In our study, where raltegravir was dosed 400 mg twice daily, we did not observe any significant associations between raltegravir secondary PK parameters (C_12_ or AUC_0–12_) and time to VF or change in CD4 from baseline, possibly due to the substantial interindividual variability observed. PK sampling was performed at single time points, 4 and 24 weeks post-therapy initiation. Due to marked variability and potential changes in adherence over time, the calculated PK parameters may not be appropriate for association with a 96-week PD endpoint. Studies using PK sampling over multiple longitudinal time points could potentially overcome these complications and help establish a clearer PK/PD relationship of raltegravir [31].

AUC_0–12_ was directly proportional to ≥grade 2 triglycerides seen in 9.2% of the patients by week 96 and to our knowledge, this is the first time this association has been observed. Raltegravir, however, is generally well-tolerated and adverse events rarely lead to treatment discontinuation [32]. Compared to other antiretrovirals, raltegravir has a favourable lipid profile, with minimal increases in total cholesterol and triglycerides [2].

Polymorphisms altering the TA repeat expansion in the TATAA box of the *UGT1A1* gene, such as UGT1A1*28 and UGT1A1*6, have been shown to influence UGT1A1 enzyme activity, resulting in changes in the PK and PD of UGT1A1 substrates. UGT1A1*6 has been reported to be associated with a higher dolutegravir C_min_ and UGT1A1*6 and UGT1A1*28 with a higher incidence of neuropsychiatric adverse events in those receiving dolutegravir [33]. UGT1A1*28 linked to increased toxicity of the anti-cancer drug irinotecan, is also well documented [34].

Studies investigating the influence of *UGT1A1* polymorphisms on raltegravir have produced mixed results. The first study to investigate this association (*n* = 57) demonstrated an elevation in *C*_min_ (91%) in patients with the UGT1A1*28/*28 genotype compared to UGT1A1*1/*1 [14]. Similarly, work conducted by Belkhir et al. (*n* = 104) observed higher raltegravir exposure and lower glucuronoconjugation rate in UGT1A1*28 carriers compared to UGT1A1*1 [13]. However, several other studies failed to show any influence of UGT1A1*28 on raltegravir PK [10, 16, 17]. Our study did not demonstrate a significant relationship between raltegravir PK and the genetic polymorphisms studied. The influence of *SLC22A6* and *UGT1A1* genotypes on CL/F did not fulfil the criteria for inclusion in the popPK model, although patients homozygous for UGT1A1*28 had 21% lower typical value of CL/F, corresponding to slightly higher AUC_0–12_ (GMR: 1.34) and C_12_ (GMR: 1.32), compared to combined low/reduced activity genotype. The impact of this polymorphism, however, was more pronounced on VF with a significantly lower incidence in those homozygous for UGT1A1*28 (*p* = 0.012), even after adjusting for baseline CD4, although clinical consequence may be questionable given that the association was lost when adjusted for both baseline CD4 cell count and HIV-1 viral load. To our knowledge, this was the first study to assess the influence of UGT1A1*28 on integrase resistance mutations and we did not see significant associations. Our findings suggest that a high intraindividual variability in raltegravir PK may mask the effects of genetic polymorphisms on single drug concentration profiles, reinforcing the need for PK investigations using multiple longitudinal time points.

A new film-coated tablet containing 600 mg of raltegravir with optimised exposure and bioavailability has been evaluated in the ONCEMRK study that demonstrated the non-inferiority of a once-daily 1200 mg (two 600 mg tablets) raltegravir-containing regimen to the standard regimen of 400 mg twice daily for initial therapy in terms of efficacy and safety [5, 35]. HIV suppression was similar in both groups despite a significant difference in median *C*_min_ concentrations: 113 nM (IQR 63–211) for 1200 mg once daily versus. 543 nM (309–1135) for 400 mg twice daily [36]. UGT1A1*28 could have a similar influence on the PK and PD of the new formulation, which needs to be investigated.

There are several limitations to our study. Raltegravir PK has been shown to be influenced by the fat content of food through a change in gastric pH, however, the clinical relevance of this interaction is questionable [37]. In our study, we did not have the data to investigate this association. Furthermore, the limited sampling scheme of one sample per patient within a dosing interval necessitating the use of priors may have influenced the parameter estimates. Although popPK is the preferred method for dealing with sparse data the prior subroutine was implemented in order to allow partition of the random effects. Indeed it has been suggested from studies in mice that at least two samples within a dosing interval are needed to adequately estimate random effects [38] (i.e. separate interindividual and residual variability). Furthermore, the available priors from the literature may not be informative for the study population and a degree of model misspecification was evident particularly during the absorption phase where data was sparsest. Despite the limitations, it is important to note that priors were not used for estimation of raltegravir CL/F (the main parameter of interest and from which AUC_0–12_ was derived), the model described the central tendency of raltegravir concentrations well and parameter estimates were consistent with literature values, providing confidence in the model and predictions.

In conclusion, there were no significant correlations between the PK and PD of raltegravir. The influence of UGT1A1*28 was more profound on the incidence of VF than on raltegravir PK, possibly masked by intraindividual variability. These findings emphasise the importance of including multiple longitudinal time points while evaluating PK/PD relationships and investigating genetic associations on raltegravir PK.

## Methods

### Patients and pharmacokinetic sampling

Between August 2010 and September 2011, 805 HIV-infected, treatment-naïve males and non-pregnant females were enroled into NEAT 001/ANRS 143 (NCT01066962), a randomised, open-label trial, from 78 clinical sites across 15 European countries. Recruitment criteria have been detailed previously [20]. Briefly, patients without any major IAS-USA resistance mutations with plasma HIV viral load >1000 copies/mL and CD4 count below 500 cells/mm^3^, unless presenting a symptomatic HIV infection were suitable to participate in the study. Patients with abnormal laboratory results, hepatic or renal insufficiency or suffering from co-infections (e.g. tuberculosis, hepatitis) were excluded. All patients received darunavir/ritonavir and were randomized 1:1 to raltegravir 400 mg twice daily (NRTI-sparing regimen) or tenofovir disoproxil fumarate/emtricitabine (standard regimen). In this sub-study, only patients randomised to the raltegravir arm were included (darunavir, ritonavir, tenofovir disoproxil fumarate and emtricitabine are presented separately) [39]. Single blood samples were taken at week 4 and 24 to obtain plasma for drug measurement. Raltegravir plasma concentrations were determined by a validated LC-MS/MS method [40] with a LLQ of 0.0117 mg/L.

### Ethics

NEAT 001/ANRS 143 was conducted in accordance with the Declaration of Helsinki and ethical approval was obtained locally from study sites. All study participants provided written informed consent [20].

### Genotyping

Genomic DNA was extracted from blood samples using the QI Amp DNA mini kit (Qiagen, West Sussex, UK). DNA was quantified using NanoDrop (Thermo Fisher Scientific, Wilmington, DE, USA). Genotyping was conducted using RT-PCR on a DNA Engine Chromo4 system (Bio-Rad Laboratories, Hercules, CA, USA). The PCR procedure consisted of denaturation (95 °C; 10 min), 50 cycles of amplification (92 °C; 15 s) and annealing (60 °C; 1.5 min) [41]. Taqman genotype master mix and assays, *SLC22A6* 453G>A (rs4149170, designed using Custom TaqMan® Assay Design Tool) and *SLC22A6* 728C>T (rs1*15*68626, C__25598602_40) were purchased from Life Technologies (Paisley, Renfrewshire, UK). Opticon Monitor software (v. 3.1, Bio-Rad Laboratories) was used to obtain allelic discrimination plots and identify genotypes. *UGT1A1* was genotyped using the Sequenom MassARRAY platform and iPLEX Pro UGT1A1-TA assays (Sequenom Laboratories, San Diego, CA, USA). Similar to methods described by Lee et al. [42], 20 ng of genomic DNA was amplified by PCR and then treated with shrimp alkaline phosphatase to inactivate unincorporated nucleotides. Using iPLEX Gold Reaction Cocktail, single base extension reaction was performed followed by spotting onto SpectroCHIP II. Data were analysed by MassARRAY TYPER software (v. 4.0.20, Sequenom Laboratories).

### Population pharmacokinetic modelling

Raltegravir plasma concentration-time data were analysed using nonlinear mixed effects (NONMEM v. 7.3, ICON Development Solutions, Ellicott City, MD, USA) with FOCE-I estimation [43]. The $PRIOR subroutine was implemented due to the sparseness of the sampling with single samples drawn per patient on two separate clinic visits, 4 and 24 weeks after therapy initiation. Parameter estimates and corresponding variances from a previous popPK analysis were used as priors [22].

Covariates including weight, age, sex, ethnicity and UGT1A1*28, *SLC22A6* 453G>A and *SLC22A6* 728C>T genotypes were primarily investigated by univariable analysis for associations with raltegravir CL/F. If covariates were significant they were progressed to multivariable analysis. Genotypes were parameterised and the common allele homozygotes were used as reference to compare heterozygotes and rare allele homozygotes. Studies have demonstrated the influence of *UGT1A1* polymorphisms on UGT1A1 activity. Studies assessing promoter activity have shown that a TA insertion to give TA_7_ (UGT1A1*28) reduces gene transcription compared to the wild type TA_6_ (UGT1A1*1) [44, 45]. TA_8_ (UGT1A1*37) repeats cause lower transcription compared to TA_7_ and TA_5_ (UGT1A1*36) cause higher transcription compared to the wild type [46]. Moreover, the UGT1A1 protein is seen to be twofold lower in UGT1A1*28/*28 compared to those having the wild type. Based on the UGT1A1 activity, the patients in this study were grouped as normal (*1/*1, *1/*36), reduced (*1/*6, *1/*28, *1/*37, *28/*36, *36/*37) and low (*28/*28, *28/*37 or *37/*37) in accordance with the Clinical Pharmacogenetics Implementation Consortium (CPIC) guidelines [46]. Missing genetic data were included as a separate fixed effect to maximise data use [47].

To distinguish the difference between nested models a decrease in the minimal OFV (−2 log likelihood) of at least 3.84 units was necessary (*p* = 0.05, χ^2^ distribution, 1 d.f.). A forwards inclusion process was used to incorporate significant covariates followed by backwards elimination, retaining the biologically plausible covariates that produced an increase in OFV of at least 10.83 units (*p* = 0.001, χ^2^ distribution, 1 d.f.). This threshold was chosen in order to vigorously test the relationships observed, given sparseness of the concentration-time data per patient.

A VPC was performed to evaluate the overall model suitability by performing 1000 simulations of the raltegravir dataset using Perl-speaks-NONMEM software (PsN; version 3.4.2) [48] and plotted with Xpose4 [49] in RStudio (version 1.1.383). The final model was used to predict raltegravir AUC_0–12_, *C*_max_, C_12_, and half-life for each patient included in the model. In addition to the popPK assessment of the relationship between raltegravir CL/F and UGT1A1*28, a post-hoc analysis was performed to evaluate the influence of *UGT1A1* polymorphisms on model predicted raltegravir AUC_0–12_ and C_12_ using geometric mean ratios (GMR) and 90% confidence intervals. The analysis was performed on log-transformed data and subsequently back-transformed and presented as linear values.

### Pharmacokinetic–pharmacodynamic analysis

The primary PD endpoint was VF interpreted as change of any element of the initial randomised regimen before week 32 due to documented inadequate virological response (defined as reductions of <1 log_10_ copies/mL in HIV-1 RNA by week 18 or HIV-1 RNA ≥ 400 copies/mL or at week 24); failure to achieve virological response ≤50 copies/mL by week 32; HIV-1 RNA of 50 copies/mL or higher at any time after 32 weeks, confirmed by a second measurement). Multivariable Cox regression was utilised to assess the association between model-predicted log_10_(C_12_) or log_10_(AUC_0–12_) and time to VF, adjusting for sex, age, mode of HIV infection, ethnicity, country, baseline CD4 + cell count, and baseline HIV-1 RNA. Various sensitivity analyses were also performed: a) censoring analysis time when any component of the initial randomised treatment was stopped; b) multiple imputation of missing PK parameters (using the same factors as described above plus the event indicator and the Nelson–Aalen estimator) [50].

Similar analyses were performed to evaluate the influence of *UGT1A1* polymorphisms on VF to week 96. As an additional PD endpoint, we investigated the relationship between change in CD4 cell count from baseline to week 96 with log_10_(C_12_) or log_10_(AUC_0–12_) using multivariable linear regression models adjusting for baseline CD4 and other factors as described above.

### Adverse events

Multivariable Cox models were used to analyse the association between model-predicted log_10_(*C*_max_) or log_10_(AUC_0–12_) with predefined adverse event endpoints, grade 2 or higher creatine kinase or triglycerides (time from randomisation to first occurrence). Generalised estimating equations (GEEs) were used to analyse the association of the same PK parameters and LDL levels post-randomisation. Lipids were measured at baseline, and then at weeks 2, 4, 8, 12, 24, 48 and 96 post-randomisation. Creatine kinase was additionally measured at weeks 32, 64 and 80. All analyses were adjusted for the corresponding laboratory value at baseline. Similar analyses were preformed to assess the association between *UGT1A1* genotypes and adverse events.

### Integrase resistance

Genotypic testing was requested in case of VF or any single VL > 500 copies/mL at or after week 32 [51]. Integrase mutations were interpreted according to the 2014 IAS-USA list of mutations [52]. Kaplan-Meier analyses and Cox regression were performed to assess the association of raltegravir PK parameters and *UGT1A1* genotypes on integrase resistance, assuming that patients who did not experience VF did not develop resistance.

## Supplementary information

Supplementary Material

## Data Availability

The data that support the findings of these analyses are available upon reasonable request from the authors and with the permission of the trial sponsor and coordinating investigator.

## Code availability

The NONMEM code for the final model describing raltegravir PK can be found in the Supplementary Material.
