# Supplementary material for: Influence of UGT1A1 and SLC22A6 polymorphisms on the population pharmacokinetics and pharmacodynamics of raltegravir in HIV-infected adults: a NEAT001/ANRS143 sub-study
Source: Pharmacogenomics J. 2022 Oct 20;23(1):14–20. doi: 10.1038/s41397-022-00293-5 (PMC9584256; doi:10.1038/s41397-022-00293-5)
Supplement: Supplementary file 1 — Supplementary Material [file 41397_2022_293_MOESM1_ESM.docx]

**Table S1** Summary of univariate analysis to determine the impact of demographics and relevant genetic polymorphisms on raltegravir apparent oral clearance (n=349 patients; n=348 for genetic analysis due to 1 patient excluded for *UGT1A1*36/*36*; Note that multivariate analysis was not possible due to no significant covariates).

| ***Univariate*** | | | | | | |
| --- | --- | --- | --- | --- | --- | --- |
| **Covariate relationship** | **Equation** | **OFV** | **ΔOFV** | **d.f. (χ^2^ *p*<0·05)** | **ΔOFV threshold** | **Significant** |
| No covariates | CL/F=θ_1_ | -524.5 |  |  |  |  |
| Weight on CL/F  Weight on V_c_/F  Weight on Q/F  Weight on V_p_/F | CL/F= θ_1_*(Weight/70)^0^·^75^  V_c_/F= θ_1_*(Weight/70)^1^  Q/F= θ_1_*(Weight/70)^0^·^75^  V_p_/F= θ_1_*(Weight/70)^1^ | -529.8 | -5.3 | 4 | -9.49 | No |
| Sex (ref: male) on CL/F | CL/F=θ_1_*(θ_2_^FEMALE^) | -526.5 | -2.1 | 1 | -3.84 | No |
| Age on CL/F (centred on median 36 years) | CL/F= θ_1_+(θ_2_*(Age-36)) | -524.7 | -1.4 | 1 | -3.84 | No |
| Ethnicity (ref: Caucasian) on CL/F | CL/F=θ_1_*(θ_2_^ASIAN^) *(θ_3_^BLACK^) *(θ_4_^OTHER^) | -525.4 | -1.0 | 3 | -7.82 | No |
| Ethnicity (ref: Caucasian/Black/Other) on CL/F (combined due to small changes in CL/F *vs.* ref) | CL/F=θ_1_*(θ_2_^ASIAN^) | -525.4 | -0.9 | 1 | -3.84 | No |
| ***Univariate (removal of patient with UGT1A1*36/*36)*** | | | | | | |
| No covariates | CL/F=θ_1_ | -523.3 |  |  |  |  |
| Weight on CL/F  Weight on V_c_/F  Weight on Q/F  Weight on V_p_/F | CL/F= θ_1_*(Weight/70)^0^·^75^  V_c_/F= θ_1_*(Weight/70)^1^  Q/F= θ_1_*(Weight/70)^0^·^75^  V_p_/F= θ_1_*(Weight/70)^1^ | -528.6 | -5.2 | 4 | -9.49 | No |
| Sex (ref: male) on CL/F | CL/F=θ_1_*(θ_2_^FEMALE^) | -525.1 | -1.8 | 1 | -3.84 | No |
| Age on CL/F (centred on median 36 years) | CL/F= θ_1_+(θ_2_*(Age-36)) | -524.7 | -1.4 | 1 | -3.84 | No |
| Ethnicity (ref: Caucasian) on CL/F | CL/F=θ_1_*(θ_2_^ASIAN^) *(θ_3_^BLACK^) *(θ_4_^OTHER^) | -524.3 | -1.0 | 3 | -7.82 | No |
| Ethnicity (ref: Caucasian/Black/Other) on CL/F (combined due to small changes in CL/F *vs.* ref) | CL/F=θ_1_*(θ_2_^ASIAN^) | -524.3 | -1.0 | 1 | -3.84 | No |
| *SLC22A6* 453G>A (ref: GG) on CL/F | CL/F=θ_1_*(θ_2_^AG^) *(θ_3_^AA^) *(θ_4_^MISS^) | -527.1 | -3.8 | 3 | -7.82 | No |
| *SLC22A6* 453G>A (ref: GG/AG) on CL/F (AG combined with GG due to small change in CL/F) | CL/F=θ_1_*(θ_2_^AA^) *(θ_3_^MISS^) | -527.1 | -3.8 | 2 | -5.99 | No |
| *SLC22A6* 728C>T (ref: CC) on CL/F (CT/TT combined due to small n numbers) | CL/F=θ_1_*(θ_2_^CT/TT^) *(θ_3_^MISS^) | -526.9 | -3.6 | 2 | -5.99 | No |
| *UGT1A1*28* (ref: NORMAL) on CL/F | CL/F=θ_1_*(θ_2_^REDUCED^) *(θ_3_^LOW^) *(θ_4_^MISS^) | -528.1 | -4.8 | 3 | -7.82 | No |
| *UGT1A1*28* (ref: NORMAL/REDUCED) on CL/F | CL/F=θ_1_*(θ_2_^LOW^) *(θ_3_^MISS^) | -527.0 | -3.6 | 2 | -5.99 | No |
| ***Final Model*** | | | | | | |
| No covariates | CL/F=θ_1_ | -524.5 |  |  |  |  |

CL/F: raltegravir apparent oral clearance; Vc/F, Vp/F: raltegravir apparent volume of distribution of the central and peripheral compartment, respectively; Q/F: intercompartmental clearance; OFV: objective function value; ΔOFV: change in objective function value; d.f.: degrees of freedom based on χ^2^ distribution (corresponds to the number of parameters added or removed from the model); ΔOFV threshold: change in OFV that must be exceeded for a significant addition/removal of parameters from the model; θ_1_: typical or reference value of CL/F or V/F; θ_2-4_: relative or fractional changes in CL/F with regards to a specific covariate in comparison to the reference CL/F (θ_1_); AG, AA, REDUCED, LOW etc, MISSING, ASIAN, BLACK, OTHER, FEMALE: indicator variables for genotypes, Asian, Black, Other ethnicity and female sex, taking the value of 1 for the presence of a specific covariate group or otherwise takes the value of 0.UGT1A1*28 genotypes: *1/*1, *1/*36 (NORMAL), *1/*28, *28/*36, *36/*37 (REDUCED), *28/*28 (LOW)

**Figure S1** Goodness of fit plots for the final raltegravir model (n=349 patients) illustrating (**a**) observed concentrations (DV) *vs.* population predictions (PRED), (**b**) DV *vs.* individual predictions (IPRED), (**c**) conditional weighted residuals (CWRES) *vs.* PRED and (**d**) CWRES *vs.* time. DV *vs.* PRED and DV *vs.* IPRED plotted on a log-log scale. The fine line describes the line of unity and the bold line the line of regression.

| (**a**) |  | (**b**) |  |
| --- | --- | --- | --- |
|  |  |  |  |
|  |  |  |  |
| (**c**) |  | (**d**) |  |

**NONMEM code for the file raltegravir model**

$PROB NEAT RAL

$INPUT ID PT=DROP AMT TIME DV II SS CMT=DROP EVID MDV RATE=DROP OCC VISIT SEX AGE ETH=DROP ETH1 ETH2 ETH3 HEIGHT=DROP WEIGHT0 BMI rs4149170=DROP rs11568626=DROP UGT1A1=DROP OAT1AG=DROP OAT1AA=DROP MISS=DROP

$DATA RAL_NEAT_dat13.CSV IGNORE=#

$SUBROUTINE ADVAN4 TRANS4

$PRIOR NWPRI NTHETA=5 NETA=5 NTHP=4 NETP=0

$ESTIMATION MAXEVALS=9999 SIG=3 PRINT=2 NOABORT METHOD=COND INTERACTION POSTHOC

$COVAR

$TABLE ID TIME AMT OCC TVCL TVV2 TVQ TVV3 TVKA CL V2 Q V3 KA K12 K23 K32 ALPHA BETA HLA HLB AUC IPRED IRES CWRES NOPRINT

ONEHEADER FILE=RAL_NEAT_NEW_TAB4.TAB

$PK

TVQ=THETA(1)

Q=TVQ*EXP(ETA(3))

TVV3=THETA(2)

V3=TVV3*EXP(ETA(4))

TVKA=THETA(3)

KA=TVKA*EXP(ETA(5))

TVV2=THETA(4)

V2=TVV2*EXP(ETA(2))

TVCL=THETA(5)

CL=TVCL*EXP(ETA(1))

K12=CL/V2

K23=Q/V2

K32=Q/V3

;halflife

SUM=K12+K23+K32

ROOT=SQRT(SUM*SUM-4*K32*K12)

ALPHA=0.5*(SUM+ROOT)

BETA=0.5*(SUM-ROOT)

HLA=LOG(2)/ALPHA

HLB=LOG(2)/BETA

S2=V2

AUC=AMT/CL

$ERROR

IPRED=F

IRES=DV-IPRED

Y=F*(1+ERR(1))

$THETA (0,8.5,) ;INITIAL ESTIMATE FOR Q

$THETA (0,113,) ;INITIAL ESTIMATE FOR V3

$THETA (0,1,) ;INITIAL ESTIMATE FOR KA

$THETA (0,223,) ;INITIAL ESTIMATE FOR V2

$THETA (0,60.2,) ;INITIAL ESTIMATE FOR CL

$OMEGA 0.1 ;INITIAL ESTIMATE FOR IIV CL - ETA1

$OMEGA 0 FIX ;INITIAL ESTIMATE FOR IIV V2 - ETA2

$OMEGA 0 FIX ;INITIAL ESTIMATE FOR IIV Q - ETA3

$OMEGA 0 FIX ;INITIAL ESTIMATE FOR IIV V3 - ETA4

$OMEGA 0 FIX ;INITIAL ESTIMATE FOR IIV KA - ETA5

;THETA PRIORS FROM ARAB-ALAMEDDINE et al

$THETA 8.5 FIX ;Q

$THETA 113 FIX ;V3

$THETA 0.21 FIX ;KA

$THETA 223 FIX ;V2

;PRIOR VARIANCES FOR THETA - SE FROM ARAB-ALAMEDDINE et al - confidence in prior estimates

$OMEGA BLOCK(4)

4.6 FIX

0.00 46.3

0.00 0.00 0.033

0.00 0.00 0.00 117

$SIGMA 0.1 ;ESTIMATE FOR EPS(1) - PROP

$SCAT OMIT

*NEAT001/ANRS143 Study Group [Asterisk (*) indicates staff who left during the trial]:*

***Trial Development Team (TDT):***

- **Belgium**: Nikos Dedes (Brussels)
- **France**: Genevieve Chene, Laura Richert (Bordeaux), Clotilde Allavena, Francois Raffi (Nantes) and Brigitte Autran (Paris)
- **Italy**: Andrea Antinori, Raff aella Bucciardini and Stefano Vella (Rome)
- **Poland**: Andrzej Horban (Warsaw)
- **Spain**: Jose Arribas (Madrid)
- **UK**: Abdel G Babiker, Marta Boffito, Deenan Pillay and Anton Pozniak (London)

***Trial Steering Committee (TSC):***

- **Belgium**: Xavier Franquet* and Siegfried Schwarze (Brussels)
- **Denmark**: Jesper Grarup (Copenhagen)
- **France**: Genevieve Chene, Aurelie Fischer*, Laura Richert, Cedrick Wallet

(Bordeaux), Francois Raffi (Nantes), Alpha Diallo, Jean-Michel Molina and Juliette Saillard (Paris)

- **Germany**: Christiane Moecklinghoff (Janssen Pharmaceuticals; Neuss) and Hans-Jurgen Stellbrink (Hamburg)
- **Italy**: Stefano Vella (Rome)
- **Netherlands**: Remko Van Leeuwen (Amsterdam)
- **Spain**: Jose Gatell (Barcelona)
- **Sweden**: Eric Sandstrom (Stockholm)
- **Switzerland**: Markus Flepp (Zurich)
- **UK**: Abdel G Babiker, Fiona Ewings*, Elizabeth C George, Fleur Hudson and Anton Pozniak (London)
- **USA**: Gillian Pearce*, Romina Quercia*, Felipe Rogatto (Gilead Sciences; Foster City, CA), Randi Leavitt and Bach-Yen Nguyen* (Merck Laboratories; Whitehouse Station, NJ).

***Independent Data Monitoring Committee (IDMC):***

- Tim Peto (Chair), Oxford, UK
- Frank Goebel, Munich, Germany
- Simone Marcotullio, Rome, Italy
- Veronica Miller, Washington DC, USA.
- Peter Sasieni, London, UK

***Trial Management Team (TMT):***

- France: Clotilde Allavena and François Raffi (Nantes)
- Italy: Stefano Vella (Rome)
- UK: Anton Pozniak (London)

**CMG-EC, INSERM U897 Coordinating Unit, Bordeaux, France:**

Geneviève Chêne, Head of coordinating CTU, Member, Bordeaux, France

Fabien Arnault*, Coordinating CTU representative, Member, Bordeaux, France

Céline Boucherie*, Bordeaux CTU representative, Observer, Bordeaux, France

Aurélie Fischer*, Coordinating CTU representative, Member, Bordeaux, France

Delphine Jean*, Bordeaux CTU representative, Observer, Bordeaux, France

Virginie Paniego*, Coordinating CTU representative, Member, Bordeaux, France

Felasoa Paraina, Bordeaux CTU representative, Observer, Bordeaux, France

Laura Richert, Coordinating CTU representative, Member, Bordeaux, France

Elodie Rouch*, Bordeaux CTU representative, Observer, Bordeaux, France

Christine Schwimmer, Coordinating CTU representative, Member, Bordeaux, France

Malika Soussi*, Bordeaux CTU representative, Observer, Bordeaux, France

Audrey Taieb*, Bordeaux CTU representative, Observer, Bordeaux, France

Monique Termote, Coordinating CTU representative, Member, Bordeaux, France

Guillaume Touzeau*, Coordinating CTU representative, Member, Bordeaux, France

Cédrick Wallet, Bordeaux CTU representative, Member, Bordeaux, France

**MRC Clinical Trials Coordinating Unit, London, UK:**

Abdel G Babiker, Trial Statistician, Member, London, UK

Adam Cursley, MRC CTU representative, Observer, London, UK

Wendy Dodds*, MRC CTU representative, Member, London, UK

Fiona Ewings*, Trial Statistician, Member, London, UK

Elizabeth C George, Trial Statistician, Member, London, UK

Anne Hoppe*, MRC CTU representative, Observer, London, UK

Fleur Hudson, MRC CTU representative, Member, London, UK

Ischa Kummeling*, MRC CTU representative, Observer, London, UK

Filippo Pacciarini*, MRC CTU representative, Observer, London, UK

Nick Paton*, MRC CTU representative, Observer, London, UK

Charlotte Russell, MRC CTU representative, Observer, London, UK

Kay Taylor*, MRC CTU representative, Observer, London, UK

Denise Ward, MRC CTU representative, Observer, London, UK

**CHIP Coordinating Unit, Copenhagen, Denmark:**

Bitten Aagaard*, CHIP CTU representative, Observer, Copenhagen, Denmark

Marius Eid, CHIP CTU representative, Observer, Copenhagen, Denmark

Daniela Gey*, CHIP CTU representative, Member, Copenhagen, Denmark

Birgitte Gram Jensen*, CHIP CTU representative, Observer, Copenhagen, Denmark

Jesper Grarup, CHIP CTU representative, Member, Copenhagen, Denmark

Marie-Louise Jakobsen*, CHIP CTU representative, Observer, Copenhagen, Denmark

Per O. Jansson, CHIP CTU representative, Member, Copenhagen, Denmark

Karoline Jensen*, CHIP CTU representative, Member, Copenhagen, Denmark

Zillah Maria Joensen, CHIP CTU representative, Observer, Copenhagen, Denmark

Ellen Moseholm Larsen*, CHIP CTU representative, Observer, Copenhagen, Denmark

Christiane Pahl*, CHIP CTU representative, Observer, Copenhagen, Denmark

Mary Pearson*, CHIP CTU representative, Member, Copenhagen, Denmark

Birgit Riis Nielsen, CHIP CTU representative, Observer, Copenhagen, Denmark

Søren Stentoft Reilev*, CHIP CTU representative, Observer, Copenhagen, Denmark

**Amsterdam Medical Center Coordinating Unit, Amsterdam, The Netherlands:**

Ilse Christ, AMC CTU representative, Observer, Amsterdam, The Netherlands

Desiree Lathouwers*, AMC CTU representative, Member, Amsterdam, The Netherlands

Corry Manting, AMC CTU representative, Member, Amsterdam, The Netherlands

Remko Van Leeuwen, AMC CTU representative, Member, Amsterdam, The Netherlands

**ANRS, Paris, France:**

Alpha Diallo, Pharmacovigilance representative, Member, Paris, France

Bienvenu Yves Mendy*, Pharmacovigilance representative, Member, Paris, France

Annie Metro*, Pharmacovigilance representative, Member, Paris, France

Juliette Saillard, Sponsor representative, Member, Paris, France

Sandrine Couffin-Cadiergues, Sponsor representative, Observer, Paris, France

**ISS, Rome, Italy:**

Anne-Laure Knellwolf*, NEAT management representative, Observer, Rome, ltaly

Lucia Palmisiano, NEAT management representative, Member, Rome, ltaly

***Local Clinical Trial Units (CTU):***

**GESIDA, Madrid, Spain:**

Esther Aznar, Cristina Barea*, Manuel Cotarelo*, Herminia Esteban, Iciar Girbau*, Beatriz Moyano, Miriam Ramirez*, Carmen Saiz, Isabel Sanchez, Maria Yllescas

**ISS, Rome, Italy:**

Andrea Binelli, Valentina Colasanti, Maurizio Massella, Lucia Palmisiano.

**University of Athens Medical School, Greece:**

Olga Anagnostou, Vicky Gioukari, Giota Touloumi.

***Study Investigators:***

- **Austria:** Brigitte Schmied (National Co-ordinating Investigator), Armin Rieger, Norbert Vetter
- **Belgium:** Stephane De Wit (National Co-ordinating Investigator), Eric Florence, Linos Vandekerckhove
- **Denmark:** Jan Gerstoft (National Co-ordinating Investigator), Lars Mathiesen
- **France:** Christine Katlama (National Co-ordinating Investigator), Andre Cabie, Antoine Cheret, Michel Dupon, Jade Ghosn*, Pierre-Marie Girard, Cécile Goujard, Yves Lévy, Jean-Michel Molina, Philippe Morlat, Didier Neau, Martine Obadia, Philippe Perre, Lionel Piroth, Jacques Reynes, Pierre Tattevin, Francois Raffi, Jean Marie Ragnaud*, Laurence Weiss, Yazdanpanah Yazdan*, Patrick Yeni, David Zucman
- **Germany:** Georg Behrens (National Co-ordinating Investigator), Stefan Esser, Gerd Fätkenheuer, Christian Hoffmann, Heiko Jessen, Jürgen Rockstroh, Reinhold Schmidt, Christoph Stephan, Stefan Unger
- **Greece:** Angelos Hatzakis (National Co-ordinating Investigator), George L Daikos, Antonios Papadopoulos, Athamasios Skoutelis
- **Hungary:** Denes Banhegyi (National Co-ordinating Investigator)
- **Ireland:** Paddy Mallon (National Co-ordinating Investigator), Fiona Mulcahy
- **Italy:** Andrea Antinori (National Co-ordinating Investigator), Massimo Andreoni, Stefano Bonora, Francesco Castelli, Antonella D’Arminio Monforte, Giovanni Di Perri, Massimo Galli, Adriano Lazzarin, Francesco Mazzotta, Carlo Torti *, Vincenzo Vullo
- **The Netherlands:** Jan Prins (National Co-ordinating Investigator), Clemens Richter, Dominique Verhagen, Arne Van Eeden*
- **Poland:** Andrzej Horban (National Co-ordinating Investigator)
- **Portugal:** Manuela Doroana (National Co-ordinating Investigator), Francisco Antunes*, Fernando Maltez, Rui Sarmento-Castro,
- **Spain:** Juan Gonzalez Garcia (National Co-ordinating Investigator), José López Aldeguer, Bonaventura Clotet, Pere Domingo, Jose M Gatell, Hernando Knobel, Manuel Marquez, Martin Pilar Miralles, Joaquin Portilla, Vicente Soriano, MariaJesus Tellez
- **Sweden:** Anders Thalme (National Coordinating Investigator), Anders Blaxhult, Magnus Gisslen
- **UK:** Alan Winston (National Coordinating Investigator), Julie Fox, Mark Gompels,

Elbushra Herieka, Margaret Johnson, Clifford Leen, Anton Pozniak, Alastair Teague, Ian Williams

***Endpoint Review Committee (ERC):***

- **Australia**: Mark Alastair Boyd (Sydney)
- **Denmark**: Jesper Grarup, Per O Jansson, Nina Friis Møller and Ellen Frøsig Moseholm Larsen (Copenhagen)
- **France**: Philippe Morlat (Bordeaux), Lionel Piroth (Dijon) and Vincent Le Moing (Montpellier)
- **Netherlands**: Ferdinand W N M Wit, chair (Amsterdam)
- **Poland**: Justyna Kowalska (Warsaw)
- **Spain**: Juan Berenguer and Santiago Moreno (Madrid)
- **Switzerland**: Nicolas J Müller (Zurich)
- **UK**: Estée Török (Cambridge), Frank Post (London) and Brian Angus (Oxford)

***Sub-study working groups:***

**Virology working group:**

Vincent Calvez (co-ordinator), Charles Boucher, Simon Collins, David Dunn (statistician), Sidonie Lambert, Anne-Geneviève Marcelin, Carlo Federico Perno, Deenan Pillay, Ellen White (statistician)

**Pharmacology and adherence working group:**

Marta Boffito (co-ordinator), Adriana Ammassari, Andrea Antinori, Wolfgang Stöhr (statistician)

**Immunology working group:**

Brigitte Autran (co-ordinator), Reinhold Ernst Schmidt, Michal Odermarsky, Colette Smith, Rodolphe Thiébaut (statistician)

**Toxicity, including co-infection working group:**

Jose Arribas (co-ordinator), Jose Ignacio Bernardino De La Serna, Antonella Castagna, Stephane De Wit, Xavier Franquet, Hans-Jackob Furrer, Christine Katlama, Amanda Mocroft (statistician), Peter Reiss

**Quality of life working group:**

Raffaella Bucciardini (co-ordinator), Nikos Dedes, Vincenzo Fragola, Elizabeth C George (statistician), Marco Lauriola, Rita Murri, Pythia Nieuwkerk, Bruno Spire, Alain Volny-Anne, Brian West

**Neurocognitive function working group:**

Hélène Amieva (co-ordinator), Andrea Antinori, Josep Maria Llibre Codina, Laura Richert, Wolfgang Stöhr (statistician), Alan Winston

**Pharmaco-economics working group:**

Francesco Castelli (co-ordinator), Marco Braggion (statistician), Emanuele Focà
